# TGF-β1 secreted by pancreatic stellate cells promotes stemness and tumourigenicity in pancreatic cancer cells through L1CAM downregulation

**DOI:** 10.1038/s41388-020-1289-1

**Published:** 2020-04-14

**Authors:** Donatella Delle Cave, Martina Di Guida, Valerio Costa, Marta Sevillano, Luigi Ferrante, Christopher Heeschen, Marco Corona, Antonio Cucciardi, Enza Lonardo

**Affiliations:** 1 Institute of Genetics and Biophysics ‘Adriano Buzzati-Traverso’ (IGB), CNR, Via Pietro Castellino 111, 80131 Naples, Italy; 2 Institute for Research in Biomedicine (IRB), The Barcelona Institute of Science and Technology, Barcelona, Spain; 3 Spanish National Cancer Research Centre, CNIO, Madrid, Spain

**Keywords:** Pancreatic cancer, Prognostic markers

## Abstract

Pancreatic stellate cells (PSCs) secrete high levels of transforming growth factor-β1 (TGF-β1) that contributes to the development of pancreatic ductal adenocarcinoma (PDAC). TGF-β1 modulates the expression of L1 cell adhesion molecule (L1CAM), but its role in tumour progression still remains controversial. To clarify L1 function in PDAC and cellular phenotypes, we performed L1CAM cell sorting, silencing and overexpression in several primary pancreatic cancer cells. PSCs silenced for *TGF-β1* were used for crosstalk experiments. We found that TGF-β1 secreted by PSCs negatively regulates L1CAM expression, through canonical TGF-β-Smad2/3 signalling, leading to a more aggressive PDAC phenotype. Cells with reduced expression of L1CAM harboured enhanced stemness potential and tumourigenicity. Inactivation of TGF-β1 signalling in PSCs strongly reduced the aggressiveness of PDAC cells. Our data provide functional proof and mechanistic insights for the tumour-suppressive function of L1CAM via reducing stemness. Rescuing L1CAM expression in cancer cells through targeting of TGF-β1 reverses stemness and bears the potential to improve the still miserable prognosis of PDAC patients.

## Introduction

Pancreatic ductal adenocarcinoma (PDAC) is the fourth-leading cause of cancer-related death in the world, with a 5-year survival rate of <5% [1]. Chemotherapy resistance and tumour relapse are two unresolved problems in PDAC treatment. Cancer stem cells (CSCs) are key drivers in tumour progression, resistance and relapse, and studying their biology may provide novel insights to overcome these problems [2]. Indeed, upregulation of detoxifying enzymes and drug transporters in pancreatic CSCs have already been identified as important mechanisms for chemoresistance [3]. Hence, targeting the CSC niche and their stemness could be a complementary therapeutic strategy against cancer.

Mutational inactivation of transforming growth factor beta (TGF-β) signalling is crucial during PDAC progression and affects 40–50% of patients [4]. Rescuing TGF-β signalling in human PDAC cells abrogates their proliferation and tumourigenicity, implying that TGF-β signalling exerts tumour-suppressive effects. While these genetic and mutational data do support a tumour suppressor role for TGF-β signalling in PDAC development, high levels of TGF-β1 in patients with PDAC are associated with poor prognosis in the clinical setting [5]. TGF-β signalling is also one of the most important features forming the CSC niche and promotes plasticity in PDAC [6]. The pancreatic stellate cells (PSC) within the tumour microenvironment represent the principal source of TGF-β1 [7, 8], but still very little is known about the TGF-β1-mediated crosstalk between PSC and PDAC cells. While L1 cell adhesion molecule (L1CAM; CD171) was originally discovered in the nervous system due to its important function for axon guidance and cell migration [9–15], it has also been shown to be a crucial factor for tumour cell dissemination and metastasis in colorectal, breast, kidney and lung cancer [16, 17]. In PDAC, the expression of L1CAM could only be detected in 2 out of 111 patients (2%), whereas 98% of the samples were reportedly L1-negative [18]. Tsutsumi et al. reported that L1CAM could be detected in 23 of 107 PDAC cases (21%) [19]. Still, extensive literature strongly suggests an association between L1CAM overexpression and perineural invasion and poor outcome in PDAC [20–24]. Here, we now demonstrate for the first time, and in contrast to above-mentioned reports, that in PDAC L1CAM acts as a tumour suppressor by specifically targeting the highly tumourigenic subpopulation of CSC, thereby rationalising at least in part the adverse outcome of patients with L1CAM-low tumours. Mechanistically, we found that TGF-β1 secreted by PSC inhibits L1CAM expression on PDAC cells, thereby counteracting the CSC suppressive activities of L1CAM and subsequently promoting a more stem-like and aggressive phenotype. These findings suggest a potentially new strategy for targeting CSC in order to alleviate PDAC progression and improve the outcome of PDAC patients.

## Results

### Increased L1CAM expression is associated with favourable outcome in PDAC

**Fig. 1:**
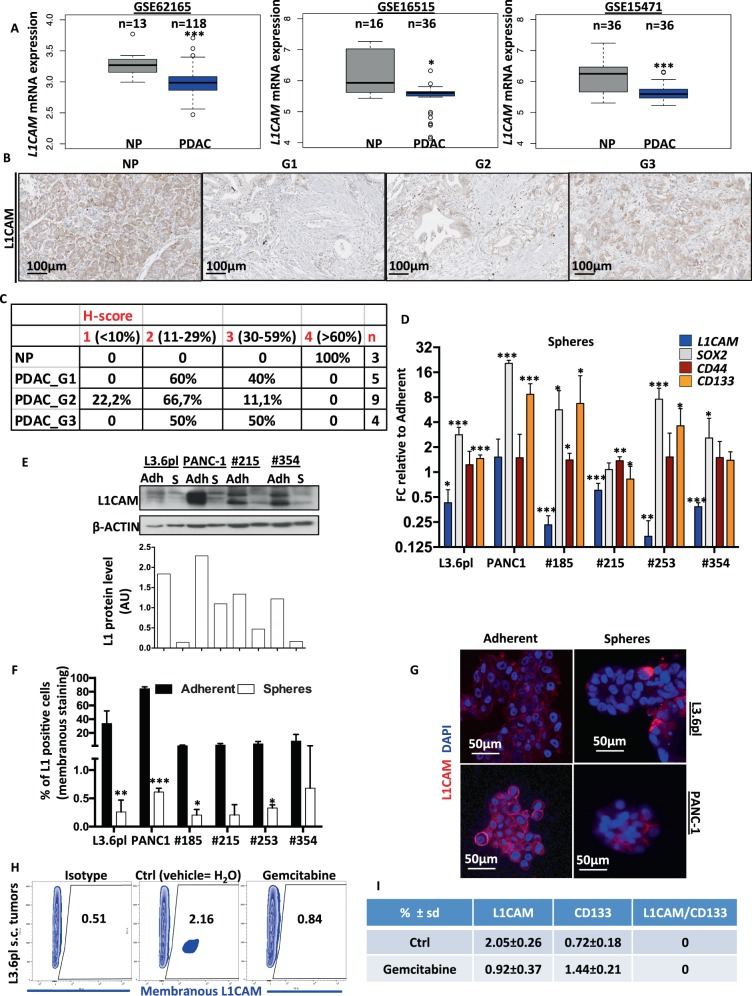
Increased L1CAM expression is associated with favourable outcome in PDAC. **a** Boxplots showing the differential expression of *L1* in PDAC samples versus normal tissue (NP) in the indicated series of transcriptomic data. *p < 0.05; ***p < 0.0005 compared with NP. **b** Immunohistochemistry for L1CAM (brown) in tissue sections from normal pancreas (P) and patients with PDAC at G1, G2 and G3 grade. **c** *H*-score for L1CAM expression. **d** qPCR analysis of *L1* and CSCs genes in adherent cells versus spheres. Data are normalised to GAPDH expression and are presented as fold change (FC) in gene expression relative to adherent cells. *p < 0.05; **p < 0.005; ***p < 0.0005 compared to Adh. *n* ≥ 6. **e** Western blot analysis for L1 in adherent cells versus spheres. Parallel β-ACTIN immunoblotting was performed and signal quantification was calculated by densitometric analysis. **f** Flow cytometry quantification for L1 in adherent cells compared to spheres. All cytometry gates were established based on isotype controls. *p < 0.05; **p < 0.005; ***p < 0.0005 compared with Adh. *n* ≥ 4. **g** Representative immunofluorescence images for L1 (red) and nuclei (blue, DAPI) of adherent cells and spheres. **h** Representative flow cytometry for L1 in subcutaneous tumours derived from L3.6pl injected cells treated with vehicle (H_2_O) or gemcitabine. All cytometry gates were established based on isotype controls. *n* ≥ 4. **I** Flow cytometry quantification for L1 and CD133 in subcutaneous tumours derived from L3.6pl injected cells treated with vehicle (H_2_O) or gemcitabine.

To examine *L1CAM* (*L1*) expression patterns in PDAC patients, we used three microarray gene profiling datasets from GEO. GSE62165, representing pancreatic cancer (*n* = 118) and normal pancreas (NP) tissue (*n* = 13), GSE16515, representing pancreatic cancer (*n* = 36) and NP tissue (*n* = 16), and GSE15471, representing pancreatic cancer (*n* = 36) and matching normal pancreatic tissue samples (*n* = 36). In all three dataset, *L1* expression was downregulated in PDAC versus adjacent NP (Fig. 1a). Interestingly, *L1* expression did not inversely correlate with tumour progression (Supplementary Fig. 1A), suggesting that *L1* downregulation is an early event. To further analyse a potential link between L1 expression and PDAC, we next performed immunohistochemistry on TMA (tissue microarray) slides composed of 18 cases of pancreatic adenocarcinoma and three NP tissues. L1CAM expression was evaluated in the tumour epithelial compartment. Figure 1b shows representative immunohistochemical (IHC) images of L1 expression in NP and PDAC. L1 expression was classified as 1–4 based on the *H*-score for each PDAC_Grade (form 1–3) (Fig. 1c). No association of L1 expression with age and gender was observed. Notably, the TMA also included one pancreatic islet cell tumour that showed high L1 cytoplasmic staining, thereby resembling L1 expression patterns in NP (Supplementary Fig. 1C). The islet cells may represent an important source for L1 expression that needs to be taking into account when scoring PDAC tissue.

We also queried the Human Protein Atlas database (https://www.proteinatlas.org/ENSG00000198910-L1CAM/pathology/pancreatic+cancer) [25]. A total of 12 patients with PDAC were classified based on L1 immunohistochemistry for staining intensity and quantity. We observed that ten samples out of 12 showed no L1 staining, whereas the remaining two samples displayed either low or medium levels of L1; five samples presented negative intensity for L1 whereas five samples have L1 weak and two have L1 moderate; the distribution of stained cells showed five samples with 0%, six samples with <25% and one sample with 25–75% (Supplementary Fig. 1D). These results indicated that L1 is expressed at high levels in NP, while its expression is reduced and rather heterogeneous in PDAC. Notably, while we observed in the TCGA database that *L1* gene expression correlated with poor prognosis in PDAC patients (Supplementary Fig. 1B), differences in survival for patients with low vs. high level of L1 in other GSE dataset (i.e. GSE50827, GSE57495, GSE71727 and GSE62452, data not shown) did not reach statistical significance.

### L1CAM expression inversely correlates with CSC content and function

As poor outcome in PDAC has been related to the CSC content [26–28], we hypothesised that downregulation of L1CAM may be associated with a more pronounced CSC phenotype. We correlated the levels of L1 (gene and protein) in cells cultured in adherent (Adh; enriched for differentiated cells) versus anchorage-independent conditions (Spheres, S; enriched for CSCs) [2]. A total of four human PDAC-derived primary cultures (#185, #215, #253, #354) [2, 29] and two established pancreatic cancer cell lines (L3.6pl and PANC-1) were analysed.

Quantitative PCR (qPCR) confirmed that *L1* gene was significantly downregulated in spheres compared with adherent cells, with the exception of PANC-1. In contrast, stemness genes (i.e. *SOX2*, *CD44* and *CD133*) were overexpressed in spheres compared with 70% confluent adherent cells (Fig. 1d), suggesting an inverse correlation. The inverse correlation was also observed by Pearson’s Correlation analysis querying three patient RNA dataset (Supplementary Fig. 1F). Consistently, spheres showed also a reduced L1 protein expression as determined by western blotting (Fig. 1e) and flow cytometry (Fig. 1f and Supplementary Fig. 1E). While L1 was hardly detectable by immunofluorescence in spheres, adherent cells displayed strong cytoplasmic and membranous L1 protein expression (Fig. 1g), suggesting a dynamic regulation depending on the cell state. To exclude the possibility that the decreased expression of L1 in spheres was related to differences in culture conditions (presence/absence of serum and plastic/no plastic), we compared adherent cells versus sphere by exchanging the media. We only observed L1 downregulation concomitantly with enrichment of CSC (i.e. in spheres and organoid-like culture grew in CSC media) (Supplementary Fig. 1G).

We next correlated the gene expression levels of *L1* with the expression levels of the stemness markers *CD44* and *CD133*. For this purpose, CD44^high^ versus CD44^low^ and CD133^high^ versus CD133^low^ cells, respectively, were isolated by FACS and mRNA was extracted to determine the *L1* expression levels for each population. CD44^low^ and CD133^low^ cells both expressed higher levels of *L1* mRNA compared to their respective positive counterparts (Supplementary Fig. 2A, B). Moreover, we tested the differentiation potential of the CSC as an important feature of their plasticity. For this purpose we cultured L3.6pl and #354 cells as spheres in the absence of serum for 7 days and then plated them in adherent conditions in the presence of 10% FBS for 4 days. By qPCR we found that expression of *L1* was reduced in spheres compared to the parental adherent cells and the levels were rescued in differentiated spheres. At the same time, expression of *ABCG2*, *CD133* and *SOX2* was significantly higher in spheres and the levels decreased in the differentiated spheres (Supplementary Fig. 2C).

Finally, we injected L3.6pl cells subcutaneously into nude mice and at 100 mm^3^ tumour size mice were randomised and challenged with 100 mg/kg of intraperitoneal gemcitabine or the vehicle (H_2_O) for 1 week (2 injections per week). Immediately after treatment mice were sacrificed, tumours were measured (Supplementary Fig. 2D), and then disaggregated and stained for L1CAM. The flow cytometry analysis (Fig. 1h) revealed both a reduction of the L1CAM+ population in gemcitabine-treated mice compared with control mice and a selection for cells with reduced L1 expression. Notably, L1 expression in tumour-derived cells from control mice was also markedly lower compared with the L3.6pl cultured in adherent condition, suggesting that the implanted L1^high^ cells lose L1 upon in vivo xenotransplantation. In contrast, the CD133+ cell population increased by ~50% in gemcitabine-treated mice compared with control mice (Fig. 1i).

### Lack of L1CAM expression features factors associated with stemness

**Fig. 2:**
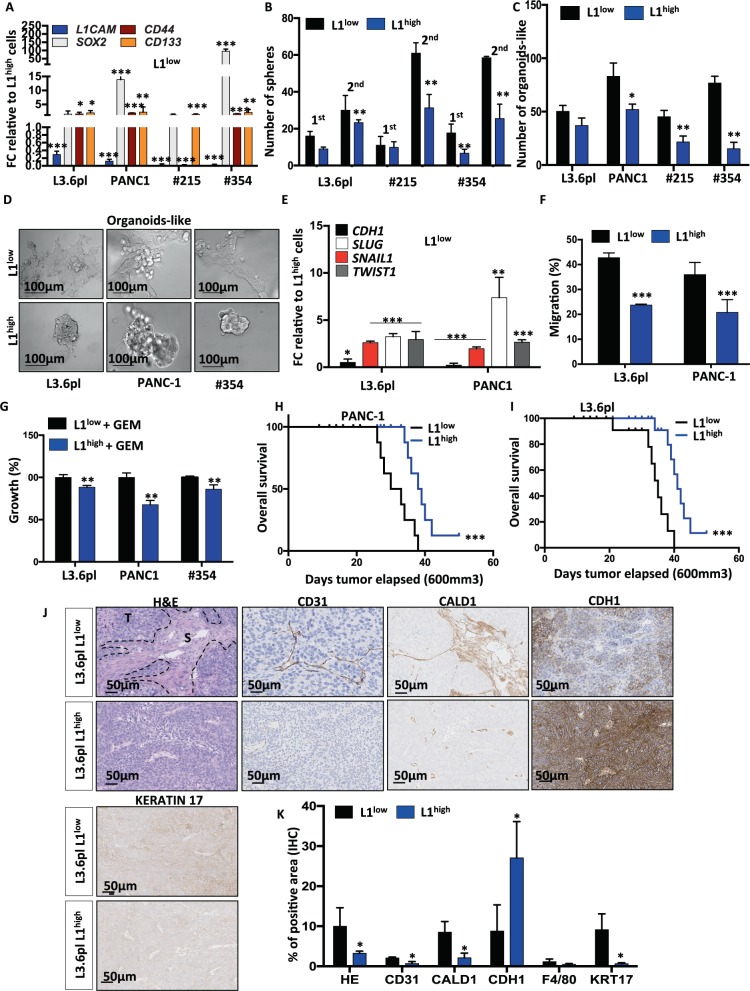
L1CAM expression inversely correlates with CSC content and function. **a** qPCR analysis for *L1* and CSCs genes in L1 sorted cells. Data are normalised to *GAPDH* and are presented as fold change in gene expression relative to L1^high^ cells. *p < 0.05; **p < 0.005; ***p < 0.0005. *n* ≥ 6. **b** Sphere formation capacity of L1 sorted cells. 200 cells per well. **p < 0.005 compared with L1^low^. *n* ≥ 6. **c** Formation of organoid-like structures of L1 sorted cells. **p < 0.005 compared with L1^low^. *n* ≥ 6. **d** Representative images of organoid-like structures derived from L1 sorted cells. **e** qPCR analysis for EMT genes in organoid-like structures derived from L1 sorted cells. Data are normalised to *GAPDH* and are presented as fold change in gene expression relative to L1^high^ cells. *p < 0.05; **p < 0.005; ***p < 0.0005. *n* ≥ 6. **f** Migratory potential of L1 sorted cells. ***p < 0.0005 compared to L1^low^. *n* ≥ 3. **g** Growth capacity of L1 sorted cells in presence of 100 μM of Gemcitabine (GEM). **p < 0.005 compared with L1^low^. *n* ≥ 6. **h**–**i** Kaplan–Meier curve of L1 sorted cells subcutaneously xenografted into athymic mice. Long-rank (Mantel–Cox) test ***p < 0.0005 compared with L1^low^. *n* = 8. **j** Representative histological sections of xenografts derived from L1 sorted cells. Tumour sections were (immuno)stained for Haematoxylin & Eosin (H&E), CD31, CALD1, E-CADHERIN (CDH1), KERATIN 17. S stroma, T tumour. **k** Quantification of fibrotic area on H&E stained sections and of CD31, CALD1, CDH1, F4/80 and KERATIN 17 detected by IHC. *p < 0.05, compared with L1^low^.

We sorted for L1CAM the L1^high^ and L1^low^ populations using L3.6pl, PANC-1, #215 and #354. The purity of the sorted cells was assessed by flow cytometry (Supplementary Fig. 2E) and qPCR (Fig. 2a). L1^low^ cells exhibited increased expression levels of stemness-related genes (i.e. *SOX2*, *CD44* and *CD133*) compared to levels in the corresponding L1^high^ cells (Fig. 2a). Notably, after 7 days in culture (Adh) the L1^high^ cells had preserved high expression level of *L1* (Supplementary Fig. 2F). To examine whether these L1^low^ cells harbour intrinsic properties of CSCs, we examined their ability to grow as spheres or as organoid-like structures. After seven days of culture, the number of the formed spheres was greater for L1^low^ compared to the corresponding L1^high^ populations (Fig. 2b and Supplementary Fig. 2G). To further explore their self-renewal capacity we trypsinised these 1st generation spheres and plated them again in ultra-low conditions for another seven days (2nd generation). Interestingly, even in the 2nd generation the L1^low^ cells had retained their higher sphere forming capacity compared with L1^high^ cells (Fig. 2b).

When cultured as single cells into matrigel [30–32] the L1^low^ cells generated more organoid-like structures than L1^high^ cells (Fig. 2c) and retained low expression of *L1* even after 7 days of culture (Org) (Supplementary Fig. 2F). The organoid-like from L1^low^ cells displayed a more invasive phenotype than the L1^high^ (Fig. 2d). Tumour cell invasion requires loss of cell–cell interactions, and is often associated with a process termed epithelial–mesenchymal transition (EMT). qPCR analysis of organoid-like (7 days old) revealed a downregulation of epithelial marker *CDH1* (E-Cadherin), whereas the mesenchymal transcription factors (i.e. *SNAIL1, SLUG* and *TWIST1*) were upregulated in L1^low^ cells compared with L1^high^ cells (Fig. 2e). Moreover we found that L1^low^ cells transmigrate more compared with L1^high^ cells (Fig. 2f). Of note, we did not observe increased proliferation in L1^low^ cells compared with L1^high^ cell, thereby excluding that the larger number of transmigrated L1^low^ cells was merely due to their enhanced proliferation (Supplementary Fig. 2H).

Then we tested the cells for chemoresistance and we found that L1^low^ cells were more resistant to gemcitabine treatment than the L1^high^ cells (Fig. 2g and Supplementary Fig. 2I). Gemcitabine is transported by multiple active nucleoside transporters (e.g. *ENT2* and *CNT3*). We found reduced expression of both *ENT2* and *CNT3* in L1^low^ compared to L1^high^ cells (Supplementary Fig. 3A). Multidrug resistance-associated proteins (MRP)s are ATP-binding cassette (ABC) pumps contributing significantly to resistance. In correlation L1^low^ cells expressed higher levels of *ABCB1*, *ABCG1* and *ABCG2*, compared with L1^high^ cells (Supplementary Fig. 3A).

Finally, we subcutaneously inoculated nude mice with L3.6pl L1^high^ or L1^low^ cells to investigate their in vivo tumourigenicity. The tumours generated from L1^low^ cells were bigger compared with those generated by L1^high^ cells (Supplementary Fig. 3B). While 250,000 L1^low^ PDAC cells were able to generate a visible tumour within few days (i.e. 9 days), the injection of L1^high^ cells resulted in a prolonged overall survival of the mice (Fig. 2h, i). By immunohistochemistry we found that the tumours generated from L1^low^ cells showed increased desmoplasia compared with the L1^high^. Specifically, we observed a higher extracellular matrix (eosin) content and augmented infiltration of endothelial cells (CD31) (Fig. 2j, k). Intriguingly, L1^low^ tumours showed an increased expression of the poor-prognosis stromal specific markers CALD1 (Fig. 2j, k). In accordance with the in vitro data the expression of the epithelial marker CDH1 was strongly reduced in the L1^low^ derived tumours compared with the L1^high^ tumours (Fig. 2j, k). We also found an increased expression of the prognostic marker KERATIN 17 [33] in the L1^low^ tumours (Fig. 2j, k). Besides, we did not observe any differences in macrophage (F4/80) infiltration, in proliferation (Ki67) and apoptosis (CASPASE-3) (Fig. 2k and Supplementary Fig. 3C). However, when assessing the expression of L1 by IHC (Supplementary Fig. 3C) or qPCR (Supplementary Fig. 3D) in the actually formed tumours, we did not observe any significant differences between L1^high^ and L1^low^ tumours. Expression of stemness genes also did not differ (data not shown). Collectively, these results can be rationalised in two ways: (1) The L1^low^ cells display enhanced tumorigenic potential compared with their L1^high^ counterparts and are able to recapitulate the original tumour heterogeneity; or (2) The L1^high^ cells (being low in CSC content) can grow tumours that have the same percentage of CSC as L1^low^ cells (rich in CSC), suggesting that the CSC state is not a fixed state, but rather dynamic.

### Knockdown of *L1CAM* promotes stemness in PDAC

**Fig. 3:**
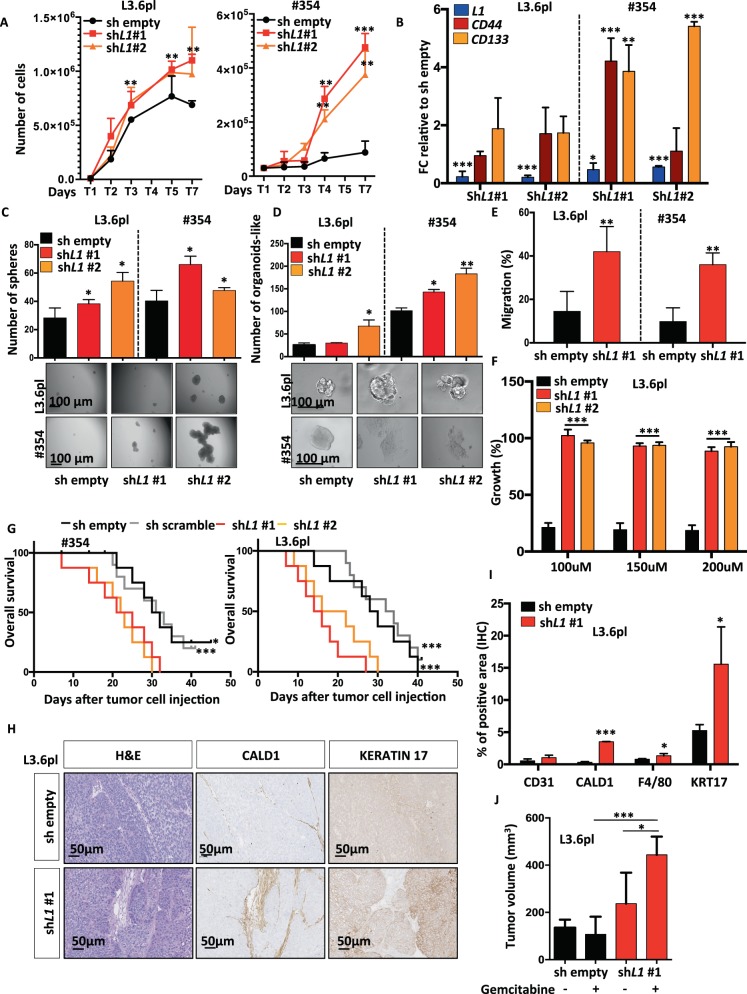
Knockdown of L1CAM promotes stemness in PDAC cells. **a** Cell expansion curves for control and *L1* knockdown cells. Cell numbers were determined daily by haemocytometer for 7 days. Each data point represents the mean ± SD of three independent experiments. **p < 0.005; ***p < 0.0005 compared to sh empty. *n* ≥ 6. **b** qPCR analysis for *L1* and CSCs of the control and *L1* knockdown cells. Data are normalised to *GAPDH* expression and are presented as fold change in gene expression relative to sh empty. *p < 0.05; **p < 0.005; ***p < 0.0005. *n* ≥ 6. **c** Sphere formation capacity of control and *L1* knockdown cells. *p < 0.05 compared with sh empty. *n* ≥ 6. **d** Formation of organoid-like structures of control and *L1* knockdown cells. *p < 0.05; **p < 0.005 compared with sh empty. *n* ≥ 6. **e** Migratory potential of control and *L1* knockdown cells. **p < 0.005 compared with sh empty. *n* ≥ 6. **f** Growth capacity of control and *L1* knockdown cells in presence of 100–150–200 μM of Gemcitabine (GEM). ***p < 0.0005 compared with sh empty. *n* ≥ 6. **g** Kaplan–Meier curve of control (sh scramble and sh empty) and sh*L1* cells subcutaneously xenografted into athymic mice. Long-rank (Mantel–Cox) test *p < 0.05 and ***p < 0.0005 compared with sh control. *n* = 8. **h** Representative histologic sections of xenografts derived from sh empty and sh*L1*#1. The tumour sections were (immuno)stained for H&E, CALD1 and KERATIN 17. **i** Quantification of CD31, CALD1, F4/80 and KERATIN 17 detected by IHC. *p < 0.05; ***p < 0.0005 compared with sh empty. **j** Tumour volume of L3.6pl cells sh empty and sh*L1*#1 subcutaneously injected into athymic mice and treated with vehicle (H_2_O) or 100 mg/Kg of Gemcitabine. *p < 0.05; ***p < 0.0005. *n* ≥ 6.

Next, we silenced *L1* in L3.6pl and #354 using two different lentiviral shRNA constructs (sh*L1*) (Supplementary Fig. 4A). Upon knockdown of *L1*, we observed an increased number of cells over time (Fig. 3a), but no morphological changes were detected in cells cultured on plastic (Supplementary Fig. 4B). We did not notice any changes in cell cycle status (Supplementary Fig. 4C) or apoptosis (Supplementary Fig. 4D).

By qPCR we observed that the sh*L1* cells exhibited increased mRNA levels of *CD44* and *CD133* compared with mock-infected cells (shEmpty) (Fig. 3b). Consistently, we found that the number of both spheres (Fig. 3c) and organoid-like (Fig. 3d) was significantly increased in sh*L1* cells compared with shEmpty. Phenotypically the sh*L1* organoid-like were more invasive (Fig. 3d lower panel) and EMT-like (Supplementary Fig. 4E) compared with shEmpty. Transmigration assays confirmed that sh*L1* cells transmigrated faster compared with shEmpty (Fig. 3e). We then tested their chemoresistance by treating L3.6pl shEmpty or sh*L1* with increased doses of gemcitabine and found a pronounced chemoresistance for sh*L1* cells compared with shEmpty (Fig. 3f).

Then, we injected 250’000 PANC-1 or L3.6pl shScramble or Empty or sh*L1* cells subcutaneously into nude mice. We observed that sh*L1* cells formed earlier (Fig. 3g) and bigger tumour (Supplementary Fig. 4F) compared with sh controls. By qPCR we proved that *L1* was still downregulated in the sh*L1* tumours and that they exhibited an upregulation in stemness genes and a marked mesenchymal phenotype (Supplementary Fig. 4G). Consistent with above data for tumours generated from L1^low^ cells, we found that tumours generated from sh*L1* cells showed increased extracellular matrix (eosin) content, expression of CALD1 and KERATIN 17 as evidenced by IHC (Figs. 3h, i). Notably, we did not observe any differences in Ki67 expression (Supplementary Fig. 4H). However, sh*L1* tumours showed a decreased L1 expression and an augmented infiltration of CD31^+^ cells and F4/80^+^ cells (Supplementary Fig. 4H and Fig. 3i). L3.6pl shEmpty or sh*L1* cells subcutaneously injected into nude mice were also treated with 100 mg/kg of gemcitabine (twice) for 1 week. Based on the tumour volume, sh*L1* cells did not grow more aggressively than shEmpty cells, but were more resistant to gemcitabine treatment (Fig. 3j).

### Ectopic overexpression of *L1CAM* inhibits stemness in PDAC

**Fig. 4:**
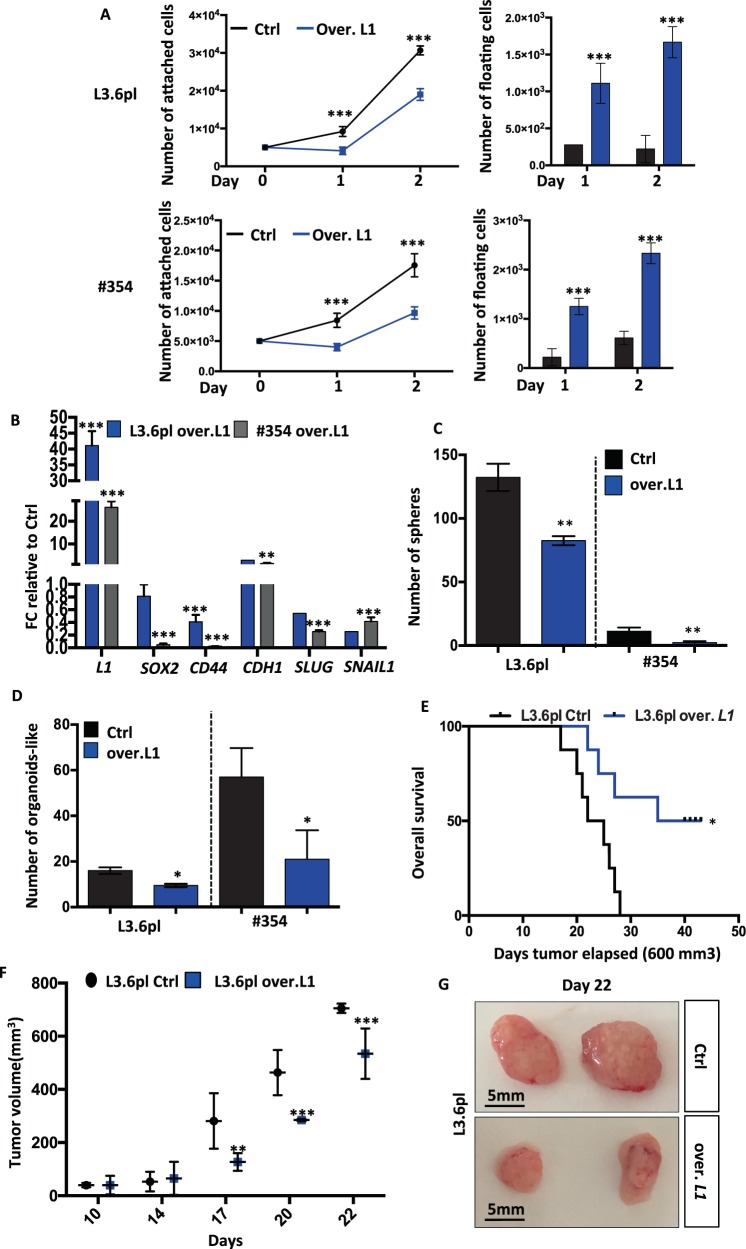
Ectopic overexpression of *L1CAM* inhibits stemness in PDAC cells. **a** Cell expansion for control and *L1* overexpressing cells. Cell viability was evaluated by trypan blue exclusion. ***p < 0.0005 compared with Ctrl. *n* ≥ 6. **b** qPCR analysis for *L1*, CSCs and EMT genes in control and *L1* overexpressing cells. Data are normalised to *GAPDH* expression and presented as fold change in gene expression relative to control cells.**p < 0.005; ***p < 0.0005. *n* ≥ 6. **c** Sphere formation capacity of control and *L1* overexpressing cells. 500 cells per well. **p < 0.005 compared with Ctrl. *n* ≥ 6. **d** Formation of organoid-like structures of control and *L1* overexpressing cells. *p < 0.05 compared with Ctrl. *n* ≥ 6. **e** Kaplan–Meier curve of control (Ctrl) and *L1* overexpressing cells subcutaneously xenografted in athymic mice. Long-rank (Mantel–Cox) test *p < 0.05 compared with Ctrl. *n* = 8. **f** Tumour volume of Ctrl and *L1* overexpressing cells subcutaneously xenografted. Data are shown as mean (points) ± s.d. (whiskers). **p < 0.005; ***p < 0.0005 compared with Ctrl. **g** Representative images at day 22 of tumours derived from control and *L1* overexpressing cells.

We constitutively overexpressed *L1* cDNA (*L1*over.)into L3.6pl, PANC-1, #253 and #354 (Supplementary Fig. 5A). *L1*over. showed a consistent reduction in cell proliferation (Supplementary Fig. 5B, C) that was particularly noticeable during the initiation phase of the cultures (day 1 and 2), most likely due to the reduced attachment capacity of *L1*over. cells (Fig. 4a and Supplementary Fig. 5D).

qPCR analysis showed that *L1*over. have a diminished expression of *SOX2* and *CD44* (Fig. 4b) as well as in a decreased capacity to form spheres and organoid-like, respectively (Fig. 4c, d). Notable, *L1*over. organoid-like have a patchier growth patterns with fewer, more compact colonies of reduced size (Supplementary Fig. 5E). Consistently, *L1*over. cells showed upregulation of *CDH1* expression and downregulation of *SLUG* and *SNAIL1* (Fig. 4b). When subcutaneously injected, 250,000 *L1*over. cells showed a significant delay in tumour elapse (Fig. 4e) and a dramatic reduction in tumour volume (Fig. 4f, g) compared with the control cells.

### PSC-derived TGF-β1 negatively regulates L1CAM expression

**Fig. 5:**
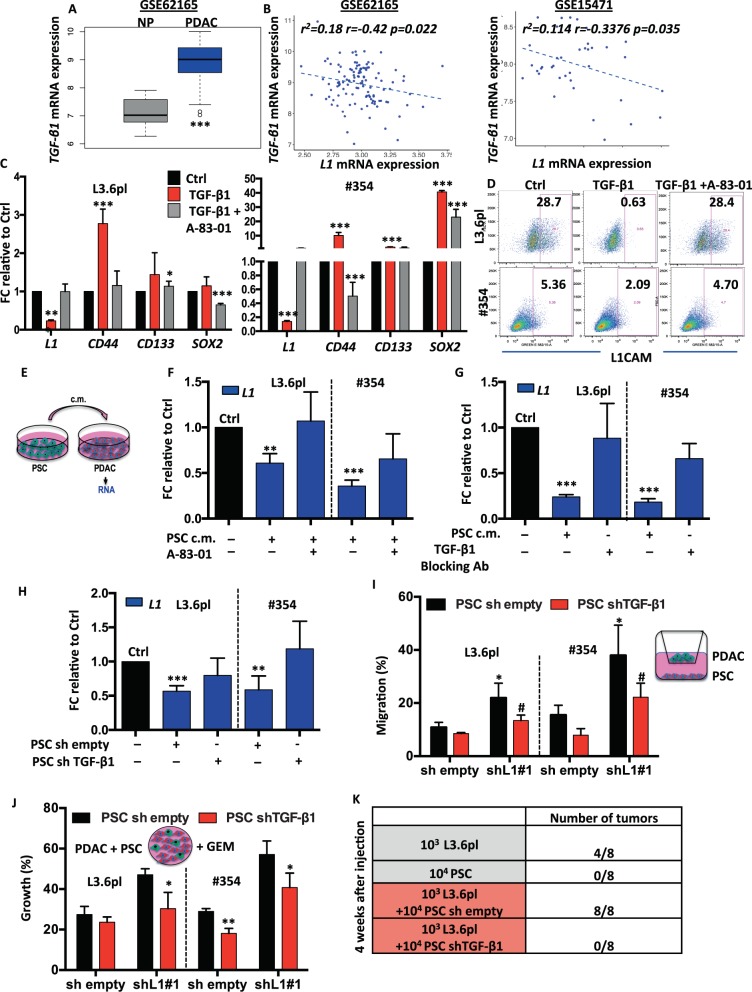
PSC-derived TGF-β1 negatively regulates L1CAM expression. **a** Boxplots showing the differential expression of *TGF-β1* in PDAC samples versus normal tissue (NP) in the indicated series of transcriptomic data. ***p < 0.0005 compared with NP. **b** Inverse correlation between *TGF-β1* and *L1* in PDAC samples in the indicated series of transcriptomic data. The *p* value is based on Pearson Correlation. **c** qPCR analysis of *L1* and CSC genes in PDAC cells untreated or treated with 10 ng/mL of recombinant TGF-β1 and 10 μM of A-83-01. Data are normalised to *GAPDH* expression and are presented as fold change in gene expression relative to Ctrl. *p < 0.05, ***p < 0.0005. *n* ≥ 6. **d** Flow cytometry for L1 in L3.6pl and #354 cells treated with 10 ng/mL of recombinant TGF-β1 and 10 μM of A-83-01 for 7 days. All cytometry gates were established based on isotype controls. *n* ≥ 3. **e** Schematic representation of the experimental design. **f** qPCR analysis of *L1* gene in PDAC cells grown in the presence of PSC conditioned medium and A-83-01. Data are normalised to *GAPDH* expression.**p < 0.005; ***p < 0.0005 compared with Ctrl. *n* ≥ 6. **g** qPCR analysis of *L1* gene in PDAC cells grown in the presence of PSC conditioned medium and TGF-β1 blocking antibody. Data are normalised to *GAPDH* expression.**p < 0.005; ***p < 0.0005 compared with Ctrl. *n* ≥ 6. **h** qPCR analysis of *L1* gene in PDAC cells grown in the presence of control or *TGF-β1* knockdown PSC conditioned media. Data are normalised to *GAPDH* expression and are presented as fold change in gene expression relative to control. **p < 0.005; ***p < 0.0005 compared with Ctrl. *n* ≥ 6. **i** Migratory potential of PDAC cells grown in the presence of control or *TGF-β1* knockdown PSC cells. *p < 0.05 compared with PSC sh empty, ^#^*p* < 0.05 compared with PSC sh empty. *n* ≥ 6. **j** Expansion capacity of PDAC cells co-cultured with control or *TGF-β1* knockdown PSC cells in the presence of 100 μM of GEM. *p < 0.05; **p < 0.005 compared with PSC sh empty. *n* ≥ 6. **k** Tumours generated from subcutaneous single injection of L3.6pl and PSC or co-injection of L3.6pl with PSC control or *TGF-β1* knockdown. *n* = 8.

We examined the mechanism regulating L1CAM expression in PDAC cells. Previous data suggested that L1CAM is directly regulated by TGF-β1 [34]. Further analysis of the GSE62165 dataset revealed that *TGF-β1* is highly expressed in PDAC compared with NP (Fig. 5a) and its expression is inversely correlated with *L1* expression in both GSE62165 and GSE15471 (Fig. 5b). We therefore hypothesised that in patients with poor prognosis, due to high levels of *TGF-β1* expression, *L1CAM* will be suppressed, resulting in unleashed stemness and consequently enhanced resistance to conventional chemotherapy. To test this hypothesis, we treated L3.6pl, #253 and #354 with recombinant TGF-β1 (rTGF-β1) protein in the presence or absence of the TGFβRI inhibitor A-83–01 for 7 days. By qPCR (Fig. 5c and Supplementary Fig. 5F) and flow cytometry (Fig. 5d and Supplementary Fig. 5F) we demonstrate a significant downregulation of L1CAM upon treatment with rTGF-β1, which was in part rescued by co-treatment with A-83–01. We also observed by qPCR an increase of stemness genes (i.e. *CD44*, *CD133* and *SOX2*) in rTGF-β1-treated cells, which was again rescued by A-83–01 (Fig. 5c and Supplementary Fig. 5G). Notably, rTGF-β1 induced p(CAGA)_12_ in L3.6pl cells, a reporter previously shown to be specifically activated by SMAD4 (Supplementary Fig. 5H). In addition, rTGF-β1 induced a time-dependent phosphorylation of SMAD2 and completely abrogated by co-treatment with A-83–01 (Supplementary Fig. 5I).

PSCs provide a supportive niche for PDAC cells, promoting their aggressiveness [35]. We found that *L1CAM* was strongly expressed in HPDE and PANC-1, but downregulated in L3.6pl, #185, #215, #253 and #354 and barely detectable in PSCs (Supplementary Fig. 5J–L). On the contrary, PSCs showed the highest expression level of *TGF-β1* (Supplementary Fig. 5J). To evaluate the paracrine effects of PSC-derived TGF-β1 on PDAC cells, we cultured PSCs in serum free medium for 3 days to collect the conditioned medium (c.m.). We then incubated L3.6pl and #354 with c.m. for 24 h (Fig. 5e). Using qPCR, we demonstrate that PSC c.m. decreased *L1* expression in PDAC cells, which was partially rescued by co-treatment with A-83–01 or a TGF-β1 blocking antibody (Fig. 5f, g and Supplementary Fig. 5M).

We genetically targeted *TGF-β1* in PSCs using a lentiviral shRNA plasmid. We repeated the crosstalk assay using shEmpty or sh*TGF-β1* PSCs c.m. While shEmpty PSC c.m. downregulated *L1* in PDAC cells, sh*TGF-β1* PSC c.m. lost the ability to downregulate *L1* expression in PDAC cells (Fig. 5h and Supplementary Fig. 5N). Functionally, we observed that sh*L1* L3.6pl and #354 cells had a more pronounced migratory phenotype in the presence of PSC c.m., both from shEmpty and sh*TGF-β1*. Moreover, the c.m. from sh*TGF-β1* PSC slightly reduced migration of both shEmpty and sh*L1* PDAC cells (Fig. 5i). Moreover we observed that the sh*L1* PDAC cells were more resistant to gemcitabine treatment in the presence of shEmpty PSCs; an effect that was significantly reduced when sh*L1* PDAC cells were co-cultured with PSC sh*TGF-β1* (Fig. 5j).

Finally we co-injected shEmpty or sh*TGF-β1* PSCs alongside L3.6pl cells into the flanks of nude mice [36]. Individual injection of 1000 L3.6pl or 10,000 PSC were used as control of the experiments. A total of 1000 L3.6pl cells co-injected with 10,000 shEmpty PSC readily formed tumours after 7 days. In contrast, 1000 L3.6pl cells co-injected with 10,000 sh*TGF-β1* PSCs failed entirely to form any tumours (0/8) during the subsequent 4 weeks of observation (Fig. 5k).

## Discussion

A better understanding of PDAC biology and tumour classification should allow us to develop more effective therapies for this deadly disease. Here, we provide comprehensive data demonstrating that downregulation of L1CAM in PDAC is a hallmark of tumour dedifferentiation and enhanced stemness. Upregulation of L1CAM counteracts the CSCs phenotype and counteracts the aggressiveness of PDAC. Therefore, L1 bears the potential as a biomarker for patient stratification and potentially also as a therapeutic target.

Using a TMA and analysing the Human Protein Atlas database we found that *L1CAM* is downregulated in the majority of pancreas carcinomas. We also noticed, exclusively in the TCGA repository, that low expression of *L1* is associated with poor outcome. Our data contrast with findings for most other cancers and previous studies on PDAC, where *L1CAM* expression was associated with poor prognosis, tumour progression and lymph nodes metastasis [37]. However, studies in a few other cancers such as childhood neuroblastoma [38], and neuroendocrine pancreatic carcinoma [18] had shown that increased *L1CAM* expression is actually linked to favourable outcome. Earlier studies in a mouse lymphoma model also demonstrated that mice bearing L1CAM positive tumours survived longer than mice bearing L1CAM negative tumours [39]. Therefore, it was of paramount importance to better understand the apparent dualistic role of L1CAM at the molecular level.

Here, we show that L1CAM^low^ PDAC cells (either L1 sorted or sh*L1*) are less differentiated and have an enhanced CSC phenotypes, including self-renewal, tumour initiation, migration, invasion and chemoresistance. Self-renewal capacity, undifferentiated state and capability to differentiate into heterogeneous population of differentiated cancer cells represent hallmarks of CSCs [40].

In PDAC, the combination of CD44 and CD133 are reasonable biomarkers for CSCs. Cells expressing these markers can also be found in secondary metastatic sites and show strong resistance to chemotherapy and radiotherapy, respectively [41]. Here, we observed that diminished levels of L1CAM correlated with a higher proportion of CD44 and CD133 cells in PDAC. Even more importantly, the ectopic downregulation of *L1CAM* increased the proportion of CD44 or CD133 cells. This effect was accompanied by increased expression of stemness-associated gene such as *SOX2*. Conversely, *L1CAM* overexpression reduced the expression of all the above-mentioned genes. Furthermore, L1^low^ cells were strongly enriched for CSC-like properties such as tumour sphere and organoid-like formation and are resistant to gemcitabine treatment. Culturing PDAC cells as organoid-like revealed that L1^low^ cells bear enhanced invasive capacity and EMT signature compared with control cells. Importantly the L1^low^ cells were able to recapitulate the tumour heterogeneity. Conversely, ectopic overexpression of *L1CAM* did not only reduce their stem-like properties and subsequently tumourigenicity, but also resulted in diminished invasiveness. Therefore, loss of the putative tumour suppressor L1CAM increases the CSC population and/or features, suggesting that L1CAM promotes epithelial (cancer) cell differentiation.

It could be argued that distinct genetic (i.e. SMAD4 status) and epigenetic features of our patient-derived PDAC cells may account for some of the discrepancies with previous reports, e.g. suggesting that L1CAM acts as oncogene in PDAC [19, 42]. Notably, however, the latter hypothesis has been difficult to align with the observation that patients with L1^low^ tumours suffer from an unfavourable outcome [18]. Our new findings may now help to solve this apparent contradiction. We propose that *L1CAM* belongs to a new type of cancer genes that can act both as an oncogene and as a tumour suppressor, depending on the cellular/genomic context. We showed that L1CAM decreases stemness, EMT and subsequently their dissemination, supporting its functional role as tumour suppressor in fully established PDAC. These data are in line with recent reports on genomic losses of *L1CAM* and hypermethylation of the *L1CAM* promoter [43].

The PDAC microenvironment is characterised by an extensive desmoplastic response, including influx of inflammatory cells, extensive deposition of collagen-rich extracellular matrix as well as activation and expansion of PSC [44]. As already reported [36], the co-injection of PSCs boosts PDAC growth and aggressiveness. In PDAC, PSCs are the predominant source of TGF-β1, which plays a critical role in tumour initiation and progression, at least in part by modulating the interactions between pancreatic cancer (stem) cells and PSC [45, 46]. We found that TGF-β/SMAD signalling is involved in the downregulation of L1CAM and concomitant maintenance of stemness. TGF-β1 secreted by PSCs strongly decreased L1CAM in PDAC cells both in vitro and in vivo. These data provide the mechanistic basis for PSCs counteracting L1CAM as a suppressor of stemness characteristics in PDAC cells. Intriguingly, Low numbers of PDAC cells co-injected with sh*TGF-β1* PSCs instead of wild type PSC completely failed to form tumours. This dramatic result underscores the pivotal role of PSC-derived *TGF-β1* in PDAC tumourigenicity.

## Materials and methods

### RNA preparation and real-time PCR

Total RNA was extracted with TRIFAST (Euroclone) according to the manufacturer’s instructions. One microgram of total RNA was used for cDNA synthesis with high-capacity reverse transcriptase (Thermofisher). Quantitative real-time PCR was performed using SYBR Green master mix (Thermofisher), according to the manufacturer’s instructions. The list of utilised primers is depicted in Table 1.

**Table 1:** List of the primer pairs used in the SYBR Green Quantitative real-time PCR.

Gene symbol	Forward primer (5′ to 3′)	Reverse primer (5′ to 3′)
*ABCB1*	TGACATTTATTCAAAGTTAAAAGCA	TAGACACTTTATGCAAACATTTCAA
*ABCG1*	TCAGGGACCTTTCCTATTCG	TTCCTTTCAGGAGGGTCTTGT
*ABCG2*	TCATGTTAAGGATTGAAGCCAAAGGC	TGTGAGATTGACCAACAGACCTGA
*CDH1*	TGCCCAGAAAATGAAAAAGG	GGATGACACAGCGTGAGAGA
*CD44*	CACGTGGAATACACCTGCAA	GACAAGTTTTGGTGGCACG
*CD133*	GCAATCTCCCTGTTGGTGAT	TCAGATCTGTGAACGCCTTG
*CNT3*	GCCGATCGTGGTTTTCTTCA	GTCATGATGGCGTGGAGTTC
*ENT2*	GAGAAGGAGCCGGAATCAGA	TTGAAGAGGAGGAAGCAGCA
*GAPDH*	CAGGAGCGAGATCCCT	GGTGCTAAGCAGTTGGT
*L1CAM*	CACTATGGCCTTGTCTGGGA	ACATACTGTGGCGAAAGGGA
*SLUG*	TTCGGACCCACACATTACCT	GCAGTGAGGGCAAGAAAAA
*SNAIL1*	CTTCCAGCAGCCCTACGAC	CGGTGGGGTTGAGGATCT
*SOX2*	AGAACCCCAAGATGCACAAC	CGGGGCCGGTATTTATAATC
*TWIST1*	AGCTACGCCTTCTCGGTCT	CCTTCTCTGGAAACAATGACAT
*TGF-β1*	AAGTGGACATCAACGGGTTC	TGCGGAAGTCAATGTACAGC

### PDAC cultures

Human immortalised PSCs and tumour-derived primary cell lines #185, #215, #253 and #354 (tissue derivation: primary pancreatic tumour; carcinoma type: PDAC) were cultured in RPMI, 10% FBS, and 50 U/ml penicillin/streptomycin [2]. tumours from patients with PDAC were obtained with written consent from all patients. The collection was performed under the Biobank of the Spanish National Cancer Research Centre (CNIO), Madrid, Spain. The human PDAC cancer cell lines L3.6pl (tissue derivation: metastatic lymph node; carcinoma type: adenosquamous carcinoma), PANC-1 (tissue derivation: pancreatic tumour; carcinoma type: ductal carcinoma) and immortalised HPDE (human pancreatic duct epithelial) cells were maintained as previously described [2]. Their identity (annually) and *Mycoplasma* free-state (bi-weekly) was routinely tested by DNA fingerprinting using short tandem repeat profiling and using the PCR-based MycoAlert Mycoplasma Detection Kit (Lonza, Bioscience), respectively. Each cell line was used for passage 4/5 after thawing from originally frozen vials.

### Suspension cultures assay

Spheres were generated and expanded in CSCs media composed of: advanced DMEM:F12 (GIBCO) supplemented with 1 × glutaMAX (GIBCO), 1 × B-27 (GIBCO), 1 × N2 (GIBCO), 20 ng/ml bFGF (basic fibroblast growth factor) (Invitrogen) and 50 ng/ml EGF (epidermal growth factor) (Peprotech). Five hundred cells per 500 µl of sphere medium were seeded in 24-well ultra-low attachment plates (Corning) as described previously [47]. After 7 days of incubation, spheres were typically >75 µm large. For serial passaging, 7-day spheres were harvested using 40 µm cell strainers, dissociated to single cells with trypsin, and then regrown for another 7 days. Cultures were kept no longer than 4 weeks after recovery from frozen stocks (passage 3–4).

### Tumour growth

All animal experiments were approved by the local ministry (IACUC protocol #992/2017-PR) and performed in the animal facility under pathogen-free conditions. Single-cell suspensions of L3.6pl cells were subcutaneously injected into 6-week-old nude CD1 male mice (Charles River Laboratories). The number of injected cells varied based on the experimental setup: 2.5 × 10^5^ of L1CAM sorted, downregulated or overexpressing cells; 1 × 10^4^ of PSC (sh empty or sh*TGFB1*) were co-injected with 1 × 10^5^ of L3.6pl. Tumour take was monitored visually and by palpation bi-weekly. Tumour diameter and volume were calculated based on calliper measurements of tumour length and width using the formula: tumour volume = (length × width^2^)/2. Tumour were considered established once length or width was >2 mm. 2.5 × 10^5^ of L3.6pl (wild type, sh empty or sh*L1*) cells were subcutaneously injected into nude mice and at 100 mm^3^ (around 10 days post injection) mice were randomised and challenged with 100 mg/kg of intraperitoneal gemcitabine or the vehicle (H_2_O) for 1 week (2 injections per week).

Further “Materials and methods” can be found as online Supplementary information.

## Conclusions

We propose a new model (Supplementary Fig. 6), in which L1CAM is negatively regulated in a paracrine fashion by *TGF-β1* secreted by PSC. Downregulation of L1CAM concomitantly leads to upregulation of stemness-associated genes and acquisition of CSC phenotypes in a subset of PDAC cells. Restoring L1CAM expression diminishes stemness and thereby sensitise tumours for chemotherapy, which should result in more lasting responses to treatment. Therefore, future studies should exploit the clinical value of these new findings.

## Supplementary information

Supplemental material

Supplementary Figure 1

Supplementary Figure 2

Supplementary Figure 3

Supplementary Figure 4

Supplementary Figure 5

Supplementary Figure 6

## Data Availability

All data that support the findings of this study are available from the corresponding authors upon reasonable request.
