# Supplementary material for: TGF-β1 secreted by pancreatic stellate cells promotes stemness and tumourigenicity in pancreatic cancer cells through L1CAM downregulation
Source: Oncogene. 2020 Apr 14;39(21):4271–85. doi: 10.1038/s41388-020-1289-1 (PMC7239770; doi:10.1038/s41388-020-1289-1)
Supplement: Supplementary file 1 — Supplemental material [file 41388_2020_1289_MOESM1_ESM.docx]

**SUPPLEMENTARY INFORMATION**

**Supplementary Figure 1 – L1CAM expression is associated with favourable outcome in PDAC and is inversely correlates with CSC content**

(**A**) Boxplots showing *L1* expression for different stages of tumour progression in PDAC samples versus normal adjacent tissue in the indicated series of transcriptomic data. (**B**) Kaplan-Meier curves showing overall survival of PDAC patients, stratified according to the median value of *L1* expression (n=84). (**C**) Representative images of L1 staining of NP and Islet cell tumour human samples. (**D**) Number of PDAC tumours (Human Protein Atlas database) classified based on L1 immunohistochemistry for staining intensity and quantity. (**E**) Flow cytometry analysis for L1 in adherent cells compared to spheres. All cytometry gates were established based on isotype controls. (**F**) Inverse correlation observed by Pearson’s Correlation analysis between L1CAM, CD133 and SOX2 in three patients RNA dataset. (**G**) qPCR analysis for *L1* in L3.6pl and #354 in different culture conditions. Data are normalized to *GAPDH* and are presented as fold change in gene expression relative to *GAPDH*. *p<0.05, ***p<0.0005 compared to condition 1. n$\geq4.$

**Supplementary Figure 2 – L1CAM expression inversely correlates with CSC content and function**

(**A**) qPCR analysis for *CD44* and *CD133* in CD44^low^ and CD133^low^ sorted cells, respectively. Data are normalized to *GAPDH* and are presented as fold change in gene expression relative to marker high cells. **p<0.005, ***p<0.0005. n$\geq4.$ (**B**) qPCR analysis for *L1* in CD44^low^ and CD133^low^ sorted cells. Data are normalized to *GAPDH* and are presented as fold change in gene expression relative to high cells. *p<0.05, ***p<0.0005. n$\geq4.$ (**C**) qPCR analysis for *L1*, *ABCG2*, *CD133* and *SOX2* in Adh *vs* Spheres *vs* Differentiated cells. Data are normalized to *GAPDH* and are presented as fold change in gene expression relative to adherent cells. *p<0.05. n$\geq4. (\mathbf{D})$Tumour volume of L3.6pl cells subcutaneously injected into athymic mice treated with vehicle (H_2_O) or 100 mg/Kg of Gemcitabine. *p<0.05, ***p<0.0005. n$\geq6.$ (**E**) Representative sorting strategy for L1 and purity control. (**F**) qPCR analysis for L1 sorted cells grown as adherent or organoids-like and analyzed after 7 days. Data are presented as fold change in gene expression relative to L1^low^ cells. (**G**) Sphere formation capacity of L1 sorted cells. **p<0.005,***p<0.0005. n$\geq6.$ (**H**) Expansion capacity of L1 sorted cells. (**I**) Expansion capacity of L1 sorted cells in presence of 150-200μM of Gemcitabine (GEM). ,***p<0.0005 compared to L1^low^. n$\geq6.$

**Supplementary Figure 3 – L1CAM expression inversely correlates with CSC content and function**

(**A**) qPCR analysis for gemcitabine resistant genes in the adherent cells derived from L1 sorted cells. Data are presented as fold change in gene expression relative to L1^high^ cells. *p<0.05, **p<0.005, ***p<0.0005. n$\geq6.$ (**B**) *In vivo* tumour growth of subcutaneously injected L1 sorted cells. Tumour size was measured every 2–5 days and tumour volume was calculated. Data are shown as mean (points) ± s.d. (whiskers). *p<0.05 compared to L1^high^ cells. n=8. (**C**) Representative histologic sections of xenografts derived from L1 sorted cells. The tumour sections were immunostained for L1CAM, F4/80, Ki67 and CASPASE-3. (**D**) qPCR analysis for L1 in tumours derived from L1 sorted cells. Data are presented as fold change in gene expression relative to *GAPDH*. n=5$.$

S**upplementary Figure 4 – Knockdown of L1CAM promotes stemness in PDAC cells**

(**A**) Flow cytometry of control and *L1* knockdown cells. All cytometry gates were established based on isotype controls. (**B**) Representative images of control and *L1* knockdown cells grown as monolayers. (**C**) Cell cycle analysis of control and *L1* knockdown cells (PI incorporation). (**D**) Flow cytometry for apoptotic cells as determined by AnnexinV/PI staining in control and *L1* knockdown cells. (**E**) qPCR analysis for EMT genes in control and *L1* knockdown cells. Data are normalized to *GAPDH* expression and are presented as fold change in gene expression relative to sh empty. *p<0.05, **p<0.005, ***p<0.0005. n$\geq6.$ (**F**) Volume of subcutaneously xenografted tumours for control and *L1* knockdown cells. Data are shown as mean (points) ± s.d. (whiskers). ***p<0.0005 compared to sh empty. n$\geq5.$ (**G**) qPCR analysis for *L1,* CSCs and EMT genes in the tumours derived from control and *L1* knockdown cells. Data are normalized to *GAPDH* expression and are presented as fold change in gene expression relative to sh empty. **p<0.005, ***p<0.0005. n$\geq6.$ (**H**) Representative histologic sections of xenografts derived from sh empty cells and sh*L1*. The tumour sections were immunostained for L1CAM, CD31, F4/80 and Ki67.

S**upplementary Figure 5– Ectopic overexpression of *L1CAM* inhibits stemness in PDAC cells**

(**A**) qPCR analysis of *L1* in Ctrl and overexpressing cells. Data are normalized to *GAPDH* expression and are presented as fold change in gene expression relative to Ctrl. ***p<0.0005. n$\geq6$. (**B-C**) Cell viability of control and *L1* overexpressing cells. Cell viability was evaluated using cell-counting-kit 8, and absorbance was measured at 450nm. *p<0.05, **p<0.005, ***p<0.0005 compared to Ctrl cells. n$\geq6.$ (**D**) Cell growth of control and *L1* overexpressing cells. Cell viability was evaluated by trypan blue exclusion. * ***p<0.0005 compared to Ctrl. n$\geq6.$ (**E**) Representative images of organoids-like derived from control and *L1* overexpressing cells. (**F**) qPCR analysis of *L1* and CSC genes in PDAC cells untreated or treated with 10ng/mL of recombinant TGF-β1 and 10μM of A-83-01. Data are normalized to *GAPDH* expression and are presented as fold change in gene expression relative to Ctrl. *p<0.05. n$\geq6.$ (**G**) Flow cytometry analysis for L1 in #253 cells treated with 10ng/mL of recombinant TGF-β1 and 10μM of A-83-01 for 7 days. All cytometry gates were established based on isotype controls. n$\geq3.$ (**H**) Luciferase activity of pCAGA12-luc SMAD4 reporter after stimulation with TGF-β1. **p<0.005 compared to Ctrl. n$\geq6$. (**I**) Western blot analysis of pSMAD2 and SMAD2 in adherent cells treated with 10ng/mL of recombinant TGF-β1 or in combination with 10μM of A-83-01. (**J**) qPCR analysis of *TGF-β1* and *L1* genes in normal pancreatic cells (HPDE), pancreatic stellate cells (PSC) and PDAC cells. Data are normalized to *GAPDH* expression. (**K**) L1 surface staining % evaluated by flow cytometry. (**L**) Western blot analysis for L1CAM and **β**-ACTIN. (**M**) qPCR analysis of *TGF-β1* in PDAC cells untreated or treated with 10ng/mL of recombinant TGF-β1 and 10uM of A-83-01 (TGF-β receptor 1 inhibitor). Data are normalized to *GAPDH* expression and are presented as fold change in gene expression relative to control.**p<0.005. n$\geq6.$ (**N**) qPCR analysis of *TGF-β1* in control or *TGF-β1* knockdown PSC cells. Data are normalized to *GAPDH* expression.***p<0.0005. n$\geq6.$

S**upplementary Figure 6 – Schematic representation of the overall mechanism of TGF-β1 downregulating L1 and subsequently driving tumour progression through stemness gene activation**

**SUPPLEMENTARY METHODS**

**Matrigel embedding culture assay.** Five hundred PDAC cells were embedded in 50µL of 100% BME2 (Cultrex) and seeded in 24-well plates (Corning). The formed spheres, here termed organoid-like structures, were cultured in CSCs medium for 7 days.

**Protein isolation and western blot analysis.** Cells were lysed with RIPA buffer (50mM Tris-HCl at pH 7.6, 150mM NaCl, 1% NP-40, 0.5% sodium deoxycholate, 0.1% SDS, 5mM EDTA plus proteases and phosphatases inhibitors) for 1hr at 4^o^C. Total protein quantification was performed with Bio-Rad Protein Assay Dye Reagent concentrate. A total of 40μg of protein was separated on 15% SDS–PAGE gels at 100 V and transferred to PVDF membranes for 2 hours at 200mA. PVDF membranes were hybridized with mouse antibodies against L1CAM (HPA005830, Sigma-Aldrich), Smad2 (#5339; Cell Signaling), β-actin (E-AB-20058, Elabscience), pSmad2 (3108; Cell Signaling), treated with peroxidase-conjugated goat anti-mouse or anti-rabbit Ig secondary antibody (DPVR-HRP, Immunologic), and then visualized by enhanced chemiluminescence (ECL Nova 2.0 XLS071, 2050 Cyanagen). n>6

**Immunofluorescence.** The cells were fixed in 4% PFA for 20 min at room temperature. After blocking with 5% bovine serum albumin in PBS-Triton 0.1%, cells were incubated with primary antibodies: L1CAM-PE (HPA005830, Sigma) overnight at 4^o^C in the dark. The nuclei of cell were stained by incubating with DAPI (Sigma). Images were acquired at room temperature using the LEICA DM6000 inverted microscope (Leica) on a DC 350 FX camera (Leica).

**Histoscore (H-score)**. The intensity of L1CAM staining was reported based on the H-score method considering the intensity and the % of positive cells. We categorised the H-score data as “1” for an H-Score <10%, “2” for 11-29%, “3” for 30-59% and “4” for >60%.

**Flow cytometry and cell sorting.** To identify CSCs anti-human membranous L1CAM-PE (A18361, Life technologies), Anti-human CD133-APC (372805, BioLegend) and anti-human CD44-APC (338805, BioLegend) were used. 7AAD (BD) was used for exclusion of dead cells. Samples (n>6) were run on the FACS Canto II (BD) and data were analysed using FlowJo 9.2 (Ashland, OR).

**Conditioned medium.** PSC were maintained in DMEM medium supplemented with 0.5% FBS and 50units/ml penicillin/streptomycin. Conditioned medium was collected three days after incubation, centrifuged and filtered prior to incubation for 24 hours with primary cancer cells. TGFB inhibition was achieved by using A-83-01 (Tocris) and TGF-beta1 antibody (MAB240, R&D).

**Plasmid construct and transfection.** L1CAM expressing plasmids and empty vectors (pcDNA3.1) were obtained from Genscript (New Jersey, USA). Transfections were performed with Lipofectamine 2000 reagent (Invitrogen, Carlsbad, USA) following the manufacturers' instructions. The pCAGA12-luc SMAD4 reporter plasmid is a synthetic SMAD responsive luciferase reporter vector that was generated by cloning 12xCAGA (consensus SMAD binding element) into the pGL3 basic plasmid (Promega). Human primary pancreatic cells were plated in 24-well tissue culture plates and co-transfected with pCAGA12-luc SMAD4 reporter and control Renilla plasmid using Lipofectamine 2000. Forty-eight hours post transfection, Gaussian luciferase and Renilla luciferase were measured using a Dual Luciferase assay kit (Promega). Luciferase activity is plotted as a percentage of the activity measured in control transfected cultures.

**Migration Assays.** Migration assays were performed using Boyden chambers (Corning). 75,000 PSC cells were added to the lower chamber and let them adhere for 24 hours. The day after, five hundred microliters of cell suspensions containing 25 000 PDAC cells were added to the inserts. The assay chambers were incubated for 22 hours at 37^o^C. Invaded cells were fixed in 4% PFA and stained with DAPI. The ratio of cells in the lower chamber versus total seeded cells was calculated.

**Cell growth and Chemoresistance assay.** Proliferation rates were determined at different time points, as reported in the figures, using the CCK8 assay kit according to the manufacturer’s instruction (Dojindo). For the chemoresistance assay the cells were treated with 100-150-200μM of Gemcitabine for 48 hours. Cell viability was determined using a CCK-8 assay kit.

**Lentiviral shRNA delivery.** As lentiviral shuttle backbone we used a pLKO shRNA plasmid (Mission SIGMA). As control we used pLKO shRNA empty expression vectors. Cells were then transduced with lentiviral particles in the presence of polybrene (8ug/ml, Sigma). The cells were seeded at a density of
30 000 cells per well in a 24-well plates and allowed to adhere overnight. The next day, the cells were infected with the lentiviral particles for 6 hours. Stably transduced cells were obtained using puromycin resistance.

**IHC in FFEPE.** Immunostainings were carried out using 4-μm tissue sections according to standard procedures. Briefly, after antigen retrieval, samples were blocked with Peroxidase-Blocking Solution (Dako, S202386) for 10 min at room temperature, and primary antibodies were then incubated with samples overnight. Slides were washed with EnVision FLEX Wash Buffer (Dako, K800721), and the corresponding secondary antibody was incubated with the sample for 45 min at room temperature. Samples were developed using 3,3′-diaminobenzidine, counterstained with hematoxylin and mounted. Antibodies against L1CAM (HPA005830, SIGMA), E-CADHERIN (610182, BD), F4/80 (eBiosciences #14‐4801), CD31 (HPA004690, SIGMA), Ki67 (MA5-14520, Thermofisher), CASPASE-3 (43-7800, Thermofisher), CALD1 (HPA008066, SIGMA), KERATIN 17 (ab53707, ABCAM) were used at 1:100 dilution overnight at 4^o^C in the dark. The nuclei of cell were stained with Haematoxylin. Images were acquired using a digital image scanning (Nanozoomer 2.0HT, Hamamatsu) and cropped using NDP.view2. The area % stain represents the ratio of the summed absolute areas of staining versus the total tissue. The area % stain was analysed by Fiji ImageJ version v3.2.28. The TMA (Biomax, PA484a) was stained following the above mentioned procedure. Images were acquired using a digital image scanning (Nanozoomer 2.0HT, Hamamatsu) and cropped using NDP.view2. The scoring algorithm takes the proportion of stained cells into consideration, as well as the intensity of the staining. The reactivity was scored in a semi-quantitative manner, which was categorized as low if less than 10% staining was observed in the epithelium; and medium or high based on the intensity if the percentage was between 10-25% and ≥25%, respectively.

**Cell cycle assay.** To synchronize the cell cultures, the cells were seeded in 6-well plate in growth medium with 10% FBS overnight. Then the cultures were rinsed by PBS and changed to serum free medium. After serum starvation for 24 hours, the cells were passaged and released into cell cycle by addition of serum. For FACS analysis, cell samples were harvested at indicated time points. Cells were trypsinised, washed in PBS, centrifuged, and pellets were fixed in 200µl of 70% ethanol and stored at -20°C until use. Cells were centrifuged and pellets resuspended in 200µl of PBS with 10µg/mL of RNAse A. Cells were incubated for 1 hour at 37°C prior to resuspension in PI. Cell-cycle analysis was carried out by flow cytometry (CANTO II). Data were analysed by DIVA software.

**Apoptosis assay.** Attached and floating cells were collected, resuspended and stained with Annexin V (550474; BD Bioscience) after incubation with Annexin V binding buffer (556454, BD PharMingen). Cells were then incubated with PI. Samples were analysed by flow cytometry using a FACS Canto II (BD), and data were analysed using DIVA Software.

**Smad2 Phosphorylation Assay.** Tumour-derived primary cells were grown for 24 hours in RPMI media supplemented with 0.5% FBS. Following starvation, cells were incubated for different times (i.e., 30 minutes, 1 hour and 24 hours) at 37°C with recombinant TGF-β1 (100-21, Peprotech) either alone or in the presence of A-83-01 (2939, Tocris). Anti-Smad2 (3103, Cell Signaling) and anti-phospho-Smad2 (3108, Cell Signaling) antibodies were used following the manufacturer’s instructions.

**Bioinformatics analysis.** Normalized expression data was downloaded from NCBI GEO (GSE62165, GSE16515 and GSE15471) with the R package GEOquery. GSE62165 consists of 118 PDAC samples and 13 control samples. GSE16515 consists of 36 tumour samples and 16 normal samples; a total of 52 samples. GSE15471 consists of 36 PDAC tumours and matching normal pancreatic tissue samples from pancreatic cancer patients. A Principal Component Analysis was performed on the entire dataset, which showed a clear separation between tumour and non-tumour samples. The expression profiles of the probes associated to the genes L1CAM (11724578_at) and TGF-β1 (11741264_x_at) were extracted and used to perform a Pearson correlation analysis with the package Hmat. A Chi-square test was performed to assign a significance to the obtained correlation values, with a threshold of 0.05. Survival was analysed using the <http://gepia2.cancer-pku.cn/#survival>. A Median Group cut-off (50% high vs 50% Low) was used. The analysis was performed considering only the classical PAAD subtype (84 patients). The Pearson’s Correlation analysis was performed using the data reported in the <http://www.analytics.pancreasexpression.org/index.php?s=icgc>. The TCGA dataset is composed by 84 patients with PDAC, the USA cohort is composed by 185 patients with PDAC and the Canadian (CA) cohort is composed by 317 patients with Pancreatic Cancer.

**Statistical Analyses.** Results for continuous variables are presented as means ± standard deviation (SD) unless stated otherwise of at least three independent experiments. Treatment groups were compared to the independent samples t test. Pair-wise multiple comparisons were performed with the one-way ANOVA (two-sided) with Bonferroni adjustment. The disease-free interval of patients was calculated using the Kaplan–Meier method, and differences among subgroups were assessed by the log-rank test. Experiments were performed a minimum of three independent times and always performed in independent triplicate samples. qPCR were repeated a minimum of five independent times in triplicate. p < 0.05 was considered statistically significant. All analyses were performed using GraphPAD Prism7. Correlation analysis were performed applying the Pearson’s correlation coefficient.
