# Supplementary figures and images for: TGF-β1 secreted by pancreatic stellate cells promotes stemness and tumourigenicity in pancreatic cancer cells through L1CAM downregulation
Source: Oncogene. 2020 Apr 14;39(21):4271–85. doi: 10.1038/s41388-020-1289-1 (PMC7239770; doi:10.1038/s41388-020-1289-1)

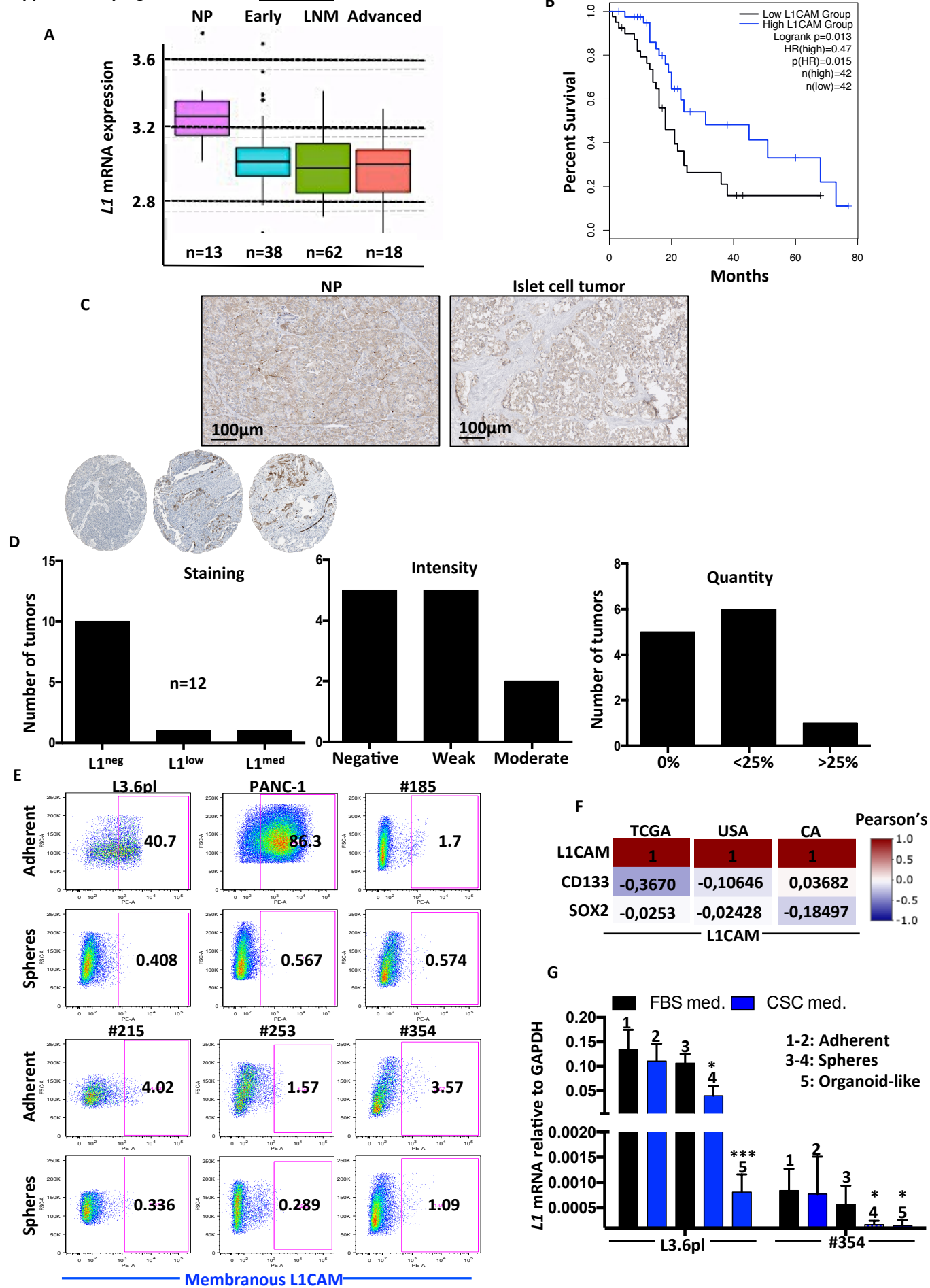

Supplement: Supplementary file 2 — Supplementary Figure 1 [file 41388_2020_1289_MOESM2_ESM.pdf]

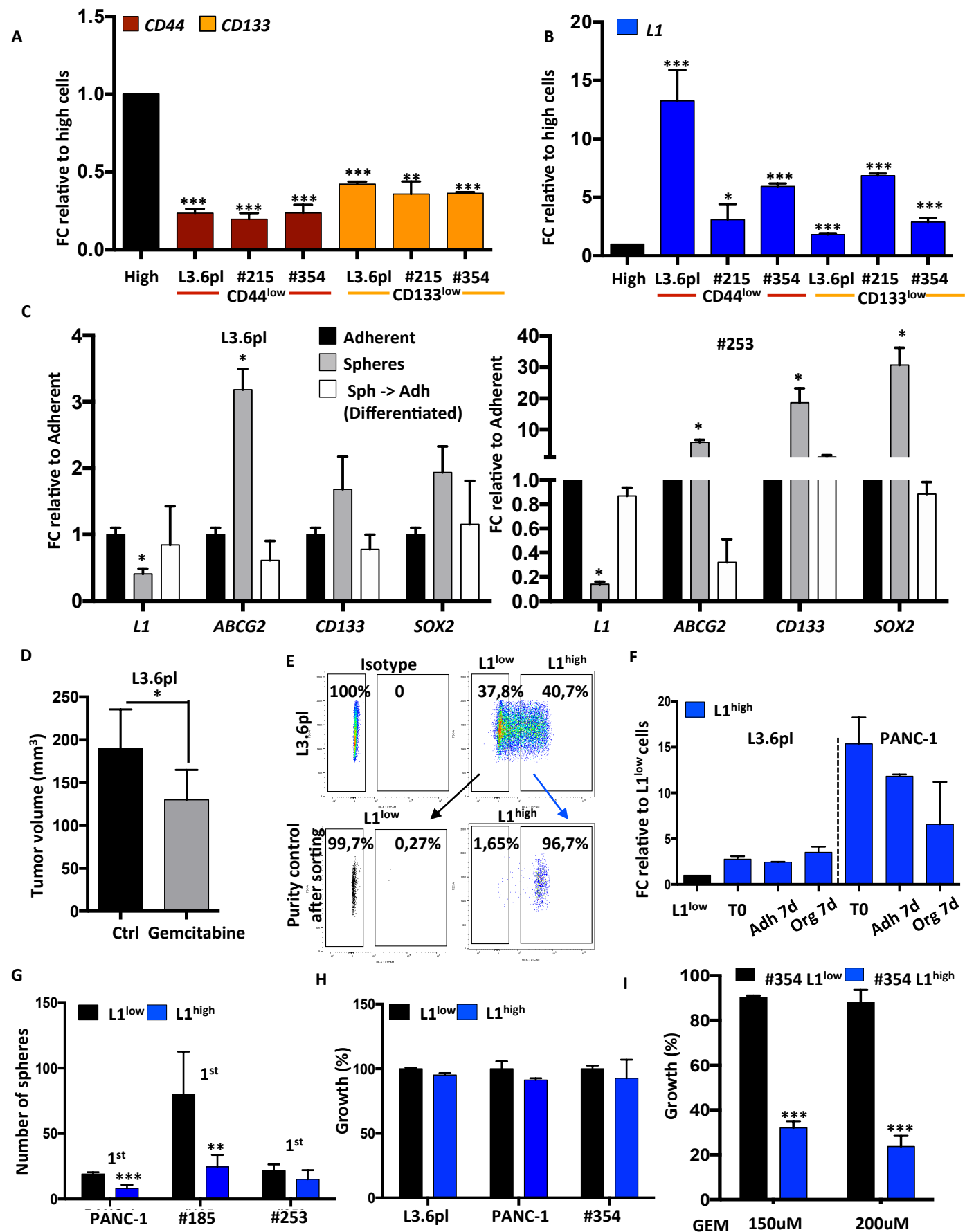

Supplement: Supplementary file 3 — Supplementary Figure 2 [file 41388_2020_1289_MOESM3_ESM.pdf]

Supplementary Figure 3

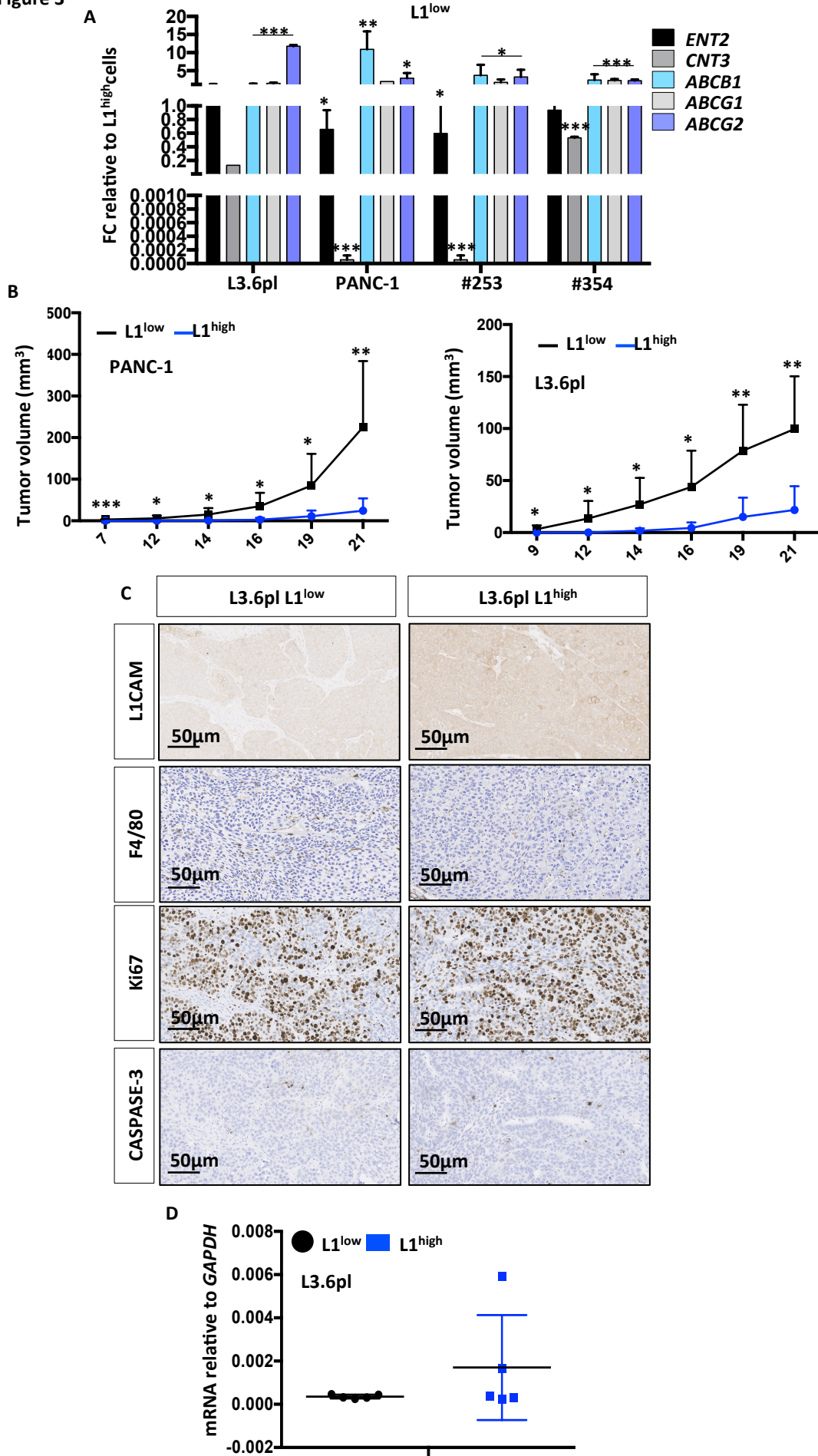

Supplement: Supplementary file 4 — Supplementary Figure 3 [file 41388_2020_1289_MOESM4_ESM.pdf]

Supplementary Figure 4

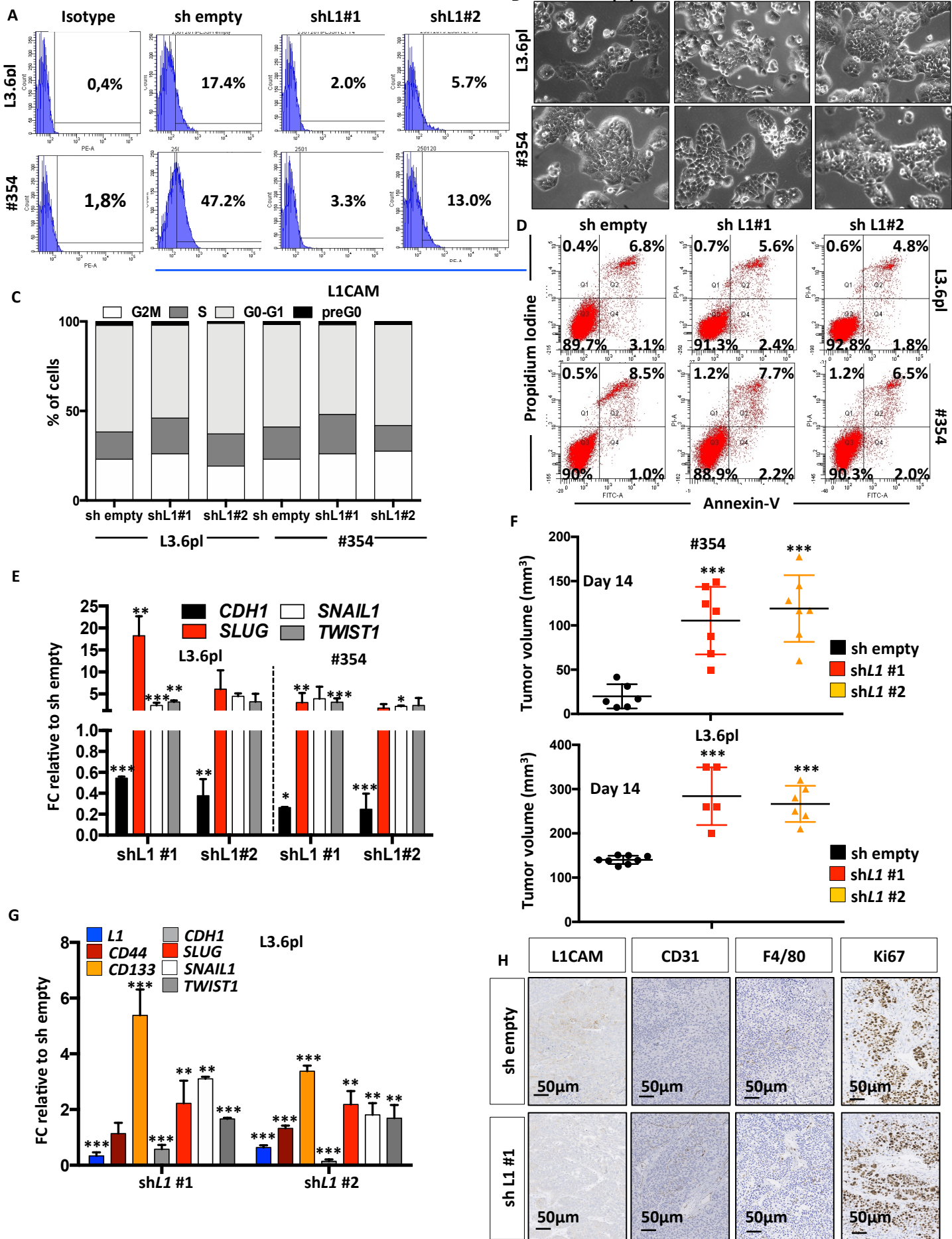

Supplement: Supplementary file 5 — Supplementary Figure 4 [file 41388_2020_1289_MOESM5_ESM.pdf]

Supplementary Figure 5

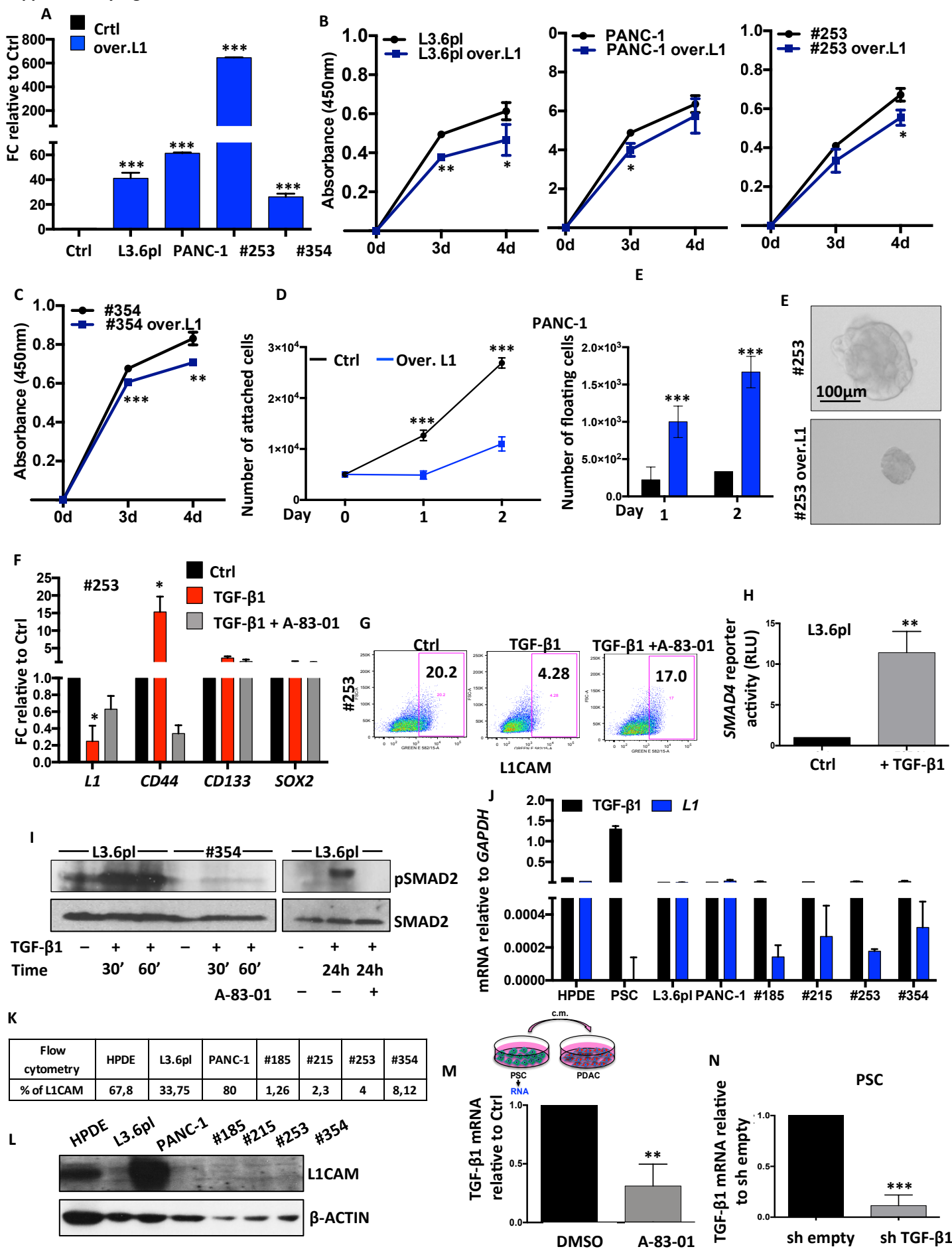

Supplement: Supplementary file 6 — Supplementary Figure 5 [file 41388_2020_1289_MOESM6_ESM.pdf]

Working model

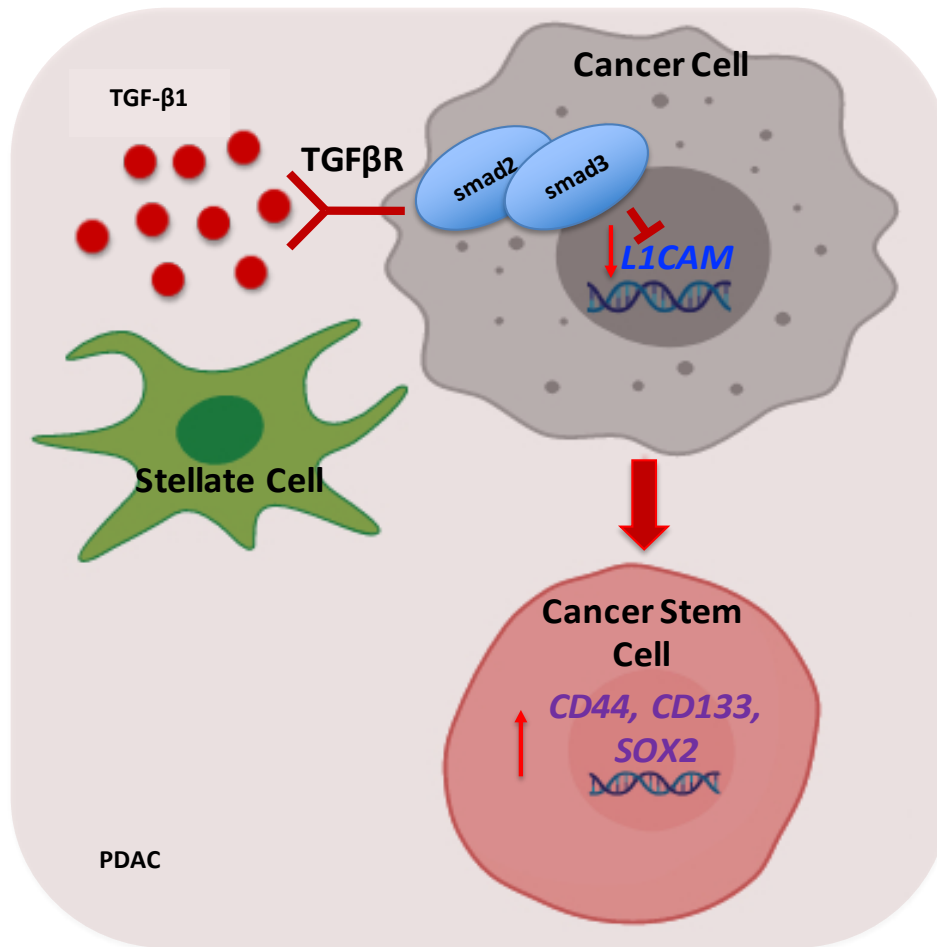

Supplement: Supplementary file 7 — Supplementary Figure 6 [file 41388_2020_1289_MOESM7_ESM.pdf]
